# Exploration of Candidate Genes Involved in the Biosynthesis, Regulation and Recognition of the Male-Produced Aggregation Pheromone of *Halyomorpha halys*

**DOI:** 10.3390/insects14020163

**Published:** 2023-02-08

**Authors:** Chunyan Wu, Feng Zhang, Youssef Dewer, Jinping Zhang, Fengqi Li

**Affiliations:** 1 National Key Laboratory of Green Pesticide, Key Laboratory of Green Pesticide and Agricultural Bioengineering, Ministry of Education, Center for R&D of Fine Chemicals of Guizhou University, Guiyang 550025, China; 2 MARA-CABI Joint Laboratory for Bio-Safety, Institute of Plant Protection, Chinese Academy of Agricultural Sciences, Beijing 100193, China; 3 Central Agricultural Pesticide Laboratory, Agricultural Research Center, Phytotoxicity Research Department, Dokki, Giza 12618, Egypt

**Keywords:** *Halyomorpha halys*, aggregation pheromone, biosynthesis, terpene synthases (TPS)

## Abstract

**Simple Summary:**

The brown marmorated stink bug, *Halyomorpha halys*, is an invasive insect pest native to Asia that was accidently introduced into North America and Europe. The male-produced aggregation pheromone of *H. halys* has great application potential in population monitoring and control of this pest. In this study, we carried out a comprehensive bioinformatics analysis to identify the candidate genes involved in the biosynthesis of the male-specific sesquiterpene aggregation pheromone of *H. halys*. We identified a novel gene, designated as *Hh*TPS1, which is related to the synthesis of the precursor molecule of the aggregation pheromone. Moreover, potential P450 reductase and transcription factors related to the aggregation pheromone biosynthesis pathway were detected. In addition, two olfactory-related genes, chemosensory protein 5 (*Hh*CSP5) and olfactory receptor 85b (*Hh*Or85b), were also identified, which are assumed to be involved in the olfactory recognition of this pheromone. Our results lay a solid foundation for further elucidating the biosynthesis of this aggregation pheromone and its behavioral regulation of *H. halys*.

**Abstract:**

The aggregation pheromone of the brown marmorated stink bug, *Halyomorpha halys* (Stål), is produced by adult males, and plays an important role in the behavioral regulation of *H. halys*. However, information on the molecular mechanisms underlying this pheromone’s biosynthesis is limited. In this study, *Hh*TPS1, a key candidate synthase gene in the aggregation pheromone biosynthesis pathway of *H. halys*, was identified. Then, through weighted gene co-expression network analysis, the candidate P450 enzyme genes in the biosynthetic downstream of this pheromone and the related candidate transcription factor in this pathway were also identified. In addition, two olfactory-related genes, *Hh*CSP5 and *Hh*Or85b, involved in the recognition of the aggregation pheromone of *H. halys*, were detected. We further identified the key amino acid sites of *Hh*TPS1 and *Hh*CSP5 that interact with substrates by using molecular docking analysis. This study provides basic information for further investigations into the biosynthesis pathways and recognition mechanisms of aggregation pheromones in *H. halys*. It also provides key candidate genes for bioengineering bioactive aggregation pheromones necessary for the development of technologies for the monitoring and control of *H. halys*.

## 1. Introduction

*Halyomorpha halys* (Stål), the brown marmorated stink bug, is a highly invasive, polyphagous insect pest. Adults and nymphs of *H. halys* damage many important crops, including fruit trees such as peaches, kiwifruit, cherries, and grapes. *H. halys* has invaded the United States, Canada, Germany, and other countries, and continues to spread worldwide [1,2]. Control of *H. halys* is mainly based on use of insecticides. However, control failure is often reported because of the rapid development of resistance by *H. halys*; therefore, safe, efficient, and more sustainable control methods should be developed [3,4].

Insect aggregation pheromones, such as that of *H. halys*, are produced by well-fed or feeding insects, and they cause hungry adults and nymphs of the same species to move to the source of the pheromone [5]. Khrimian et al. (2014) identified the two main components of the aggregation pheromones of *H. halys* as (3*S*,6*S*,7*R*,10*S*)-10, 11-epoxy-1-bisabolene-3-ol and (3*R*,6*S*,7*R*,10*S*)-10, 11-epoxy-1-bisabol-3-ol, both of which are sesquiterpene alcohols (C15) with a ratio of 3.5: 1 in adult males [6]. Lures prepared with this ratio strongly attract adults and nymphs of *H. halys* [6]. Acebes-Doria et al. (2019) confirmed that traps baited with aggregation pheromone lures were a reliable tool to monitor *H. halys* in different geographical locations and population densities throughout the growing season in the United States [7]. In addition, traps baited with aggregation pheromone lures are an effective tool for pest management decision-making [8].

Most interestingly, the *Halyomorpha halys* aggregation pheromones have also been found to attract natural enemies of this pest, as demonstrated by movement in olfactometers of two egg parasitoids. These parasitoids responded positively to volatiles from *H. halys* males but not females [9], which is consistent with the fact that only male stink bugs produce aggregation pheromones [6,10]. These findings suggest that aggregation pheromones of this pest can not only be used to trap *H. halys*, but also should attract its natural enemies [5].

Biosynthesis is an effective bioengineering technology for the synthesis of sesquiterpenes. The biosynthetic pathways of sesquiterpene production have been extensively studied in plants, bacteria, and fungi [11,12,13]. Usually, the biosynthetic pathway of sesquiterpenes is based on terpene synthase (TPS) catalyzing farnesyl diphosphate (FDP), which leads to the production of sesquiterpene backbone products or precursors. These precursors are then catalyzed by cytochrome P450 mono-oxygenase (cytochrome P450, CYP450), under the action of coenzyme CYP450 (CYP reductase, CPR) and dehydrogenases to undergo further chemical modifications that generate sesquiterpenes [14,15,16].

The biosynthetic pathway of terpenoids varies among plants, microorganisms, and insects. In 2005, Gilg et al. reported the first TPS gene from the spruce pine beetle, * Ips pini* [17]. It was found to be a trans-isoprenyl diphosphate synthases (trans-IDSs)-type TPS gene [17]. In southern green stink bug * Nezara viridula * (L.), a trans-IDS-type TPS gene was also identified. This enzyme can convert (*E,E*)-FDP to the putative pheromone precursor (+)-(*S*,*Z*)-α-bisabolene [18]. In the harlequin cabbage bug, *Murgantia histrionica* (Hahn), the aggregation pheromone was found to be very similar to the aggregation pheromones of *H. halys*. The two components of the blend in *M. histrionic* were an enantiomer mixture of (3*S*,6*S*,7*R*,10*S*)-10, 11-epoxy-1-bisabolene-3-ol and (3*R*,6*S*,7*R*,10*R*)-10, 11-epoxy-1-bisabol-3-ol [19]. Lancaster et al. (2018) identified a trans-IDS-type TPS from *M. histrionica* [20]. This TPS enzyme can convert (*E,E*)-FDP to form sesquipiperitol, which is a precursor for the aggregation pheromone [20]. These studies show that trans-IDS-type TPS is a key enzyme for the synthesis of sesquiterpene in insects.

**Figure 1:**
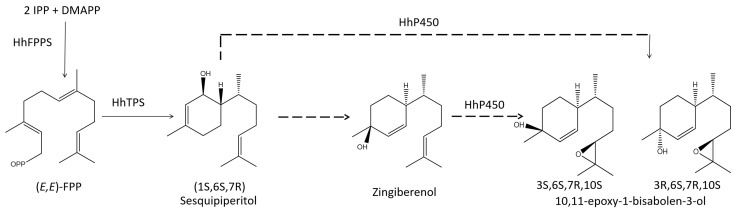
Proposed biosynthetic pathway of the aggregation pheromone in *H. halys*.

Given the similar chemical structures of the aggregation pheromones of *H. halys* and the harlequin bug, biosynthesis of aggregation pheromone in *H. halys* is also likely to be formed by TPS catalyzation of (*E,E*)-FDP, producing sesquipiperitol. Sesquipiperitol would then be catalyzed by the P450 enzyme to produce the aggregation pheromone (Figure 1). By analyzing transcriptomes of *M. histrionica* for different developmental stages and sexes, the candidate genes IDS and P450 were identified as those likely to be involved in the biosynthesis of the aggregation pheromones of this species [21]. Although the genome and transcriptome data for *H. halys* are currently available [4,22,23], the candidate genes involved in the biosynthesis of the aggregation pheromone of *H. halys* have not yet been fully explored, and transcription factors regulating the expression of these biosynthesis genes have not been reported.

The process of olfactory recognition in insects begins with the adsorption of mostly hydrophobic odorant molecules on the surface of the antenna. From here, the molecules have to pass through the pores and pore tubules of the olfactory hair cuticle to enter the sensillum. The odorant molecule has to then transverse the hydrophilic fluid filling the cuticular hair (the sensillum lymph), before reaching the dendritic membrane of the olfactory receptor neurons [24]. Detailed analyses of sensillum lymph from different insect species have led to the discovery of high concentrations of soluble proteins that bind hydrophobic compounds, including pheromones, as well as general odorant molecules. These small globular proteins have been already classified as odorant/pheromone-binding proteins (O/PBPs), chemosensory proteins (CSPs), and Niemann–Pick-type C2 proteins (NPC2), among others. When the solubilized odorant binds to odorant receptors in the dendritic membrane of the olfactory receptor cell, the cell is depolarized and generates action potentials that transmit the olfactory signal to the antennal lobe, eventually stimulating the corresponding physiological or behavioral responses [24,25,26]. Although several olfactory genes have previously been described from *H. halys*, such as CSP, OBP and OR [27,28], other olfactory genes may regulate the recognition of aggregation pheromones in *H. halys* that have not yet been reported.

In this study, diverse bioinformatics approaches were used to identify key enzyme-coding genes involved in the biosynthesis pathway of aggregation pheromone in *H. halys*, and the transcription factors that regulate the expression of these enzymes were analyzed. In addition, the olfactory genes that may be responsible for the recognition of aggregation pheromones in *H. halys* were also identified. This study identifies candidate genes for the production of aggregation pheromones of *H. halys*, aiding the development of new management and control technologies for *H. halys*.

## 2. Materials and Methods

### 2.1. Identification of Farnesyl Diphosphate (FDP) and Terpene Synthase (TPS) Genes in H. halys

The genomic coding sequences and protein sequences files of *H. halys* (assembly Hhal_2.0) were downloaded from NCBI. The FDP and trans-IDS-type TPS genes of *H. halys* were identified using the blastp method and the FDP and TPS sequences from seven other insect species, including *M. histrionica*, *Phyllotreta striolata* and *Ips pini*, with E values < 10^−10^. Names of IDS followed the reporting study [29]. The orthologous gene clusters between *H. halys* and harlequin bug (*M. histrionica*) were identified using the OrthoVenn2 (https://orthovenn2.bioinfotoolkits.net/, accessed on 14 February 2022) web tool [30]. Parameter settings were: E-value = 1× 10^-10^ and Inflation value = 1.5. For *M. histrionica*, the open reading frames were obtained by TransDecoder v5.5.0 [31] from the unigenes identified in transcriptome assembly (Genbank ID:GECQ00000000.1) [21]. MEGA6.0 [32] was used to compare the IDS protein sequences of *H. halys* with the IDS protein sequences of seven other insects, and to construct a maximum likelihood phylogenetic tree, with the bootstrap value set at 1000 and the cut-off value set at 50%.

Transcriptomes of 15 samples from different developmental life stages of *H. halys* (2nd instar, 4th instar), sexes (male and female), and tissues (antenna, salivary gland, principal salivary gland, and accessory salivary gland) were downloaded from the NCBI SRA database (Table S1). The raw data was processed with fastp [33], including adapter trimming and quality filtering, to obtain high-quality clear data, which were then aligned to the assembly Hhal_2.0 with HISAT2 [34]. We then calculated the count value of each gene expressed in the transcriptome with featureCount [35]. Then, we converted the count values for each gene into TPM expression values using Tbtools [36].

### 2.2. Weighted Gene Co-Expression Network Analysis

To identify co-expressed gene modules of the *Hh*TPS gene, we used the same 15 samples mentioned above. TPM values were used as standardized gene expression levels. The genes with zero variance, and genes with more than 10% missing sample material were filtered using the R (V.3.6.1) software (https://cran.r-project.org/bin/windows/base/old/, accessed on 8 February 2022) WGCNA (V.1.70-3) package [37], which builds a weighted gene co-expression network. To do this, first, the weight value was calculated using the function PicksoftThreshold, selecting the appropriate weight value and allowing the network to match the unmetered network distribution, and then selecting the optimal soft valve according to the analysis results. An automatic block-wise multiple-set network construction module [37] was used to build a co-expression network. We set the parameters at minmodulesize = 30, maxBlockSize = 10,000, and other parameters according to the default settings. For the co-expressed genes identified in this way, gene function annotation and enrichment analyses were performed using the KOBAS online tool [38], and the corrected *p*-value of < 0.01 was set as the threshold.

### 2.3. Molecular Docking of HhTPS1 and HhCSP 5 in H. halys

The amino acid sequences of *Hh*TPS1 and *Hh*CSP5 were used for blastp searches in the NCBI PDB and the RCSB PDB databases. The PDB ID: 2rah [39] and PDB ID: 2gvs [40] were selected as templates for *Hh*TPS1 and *Hh*CSP5, respectively. MODELLER 9v15 was used for protein homologous modeling [41]. The modeling results were evaluated with the Structural Analysis and Verification Server (https://saves.mbi.ucla.edu/, accessed on 14 February 2022). The PROCHECK server (http://servicesn.mbi.ucla.edu/PROCHECK/, accessed on 14 February 2022) was used for evaluating the quality of the modelled *Hh*TPS1 and *Hh*CSP5 proteins’ structures concerning energy and stereo-chemical geometry [42]. Using the ERRAT program from SAVES (Structure Analysis and Verification Server), non-bonded interactions were analyzed, and the quality factors for the modelled *Hh*TPS1 and *Hh*CSP5 proteins were recorded in percentages [43]. Compatibility of the atomic model (3D) with its amino acid sequence (1D) was determined with the help of the VERIFY-3D program from SAVES and the results were obtained (3D-1D Profile) in percentages [44]. 

The preparation protein and the small molecules were treated separately. The *Hh*TPS1, *Hh*CSP5 protein, and (*E,E*)-FDP, (3*S*,6*S*,7*R*,10*S*)-10,11-epoxy-1-bisabolen-3-ol, (3*R*,6*S*,7*R*,10*S*)-10,11-epoxy-1-bisabolen-3-ol were minimized by implementation of the CHARMM force field. Molecular docking analysis was carried out using the CDOCKER module [45]: “Top Hits” was set to “10”, “Pose Cluster Radius” was set to “0.1”, and all other parameters were set to default values. For *Hh*TPS1, the active pocket center was set according to PDB site records. For *Hh*CSP5, the active pocket center was defined from receptor cavities. The binding energy of the protein to the ligand compound was tested between *Hh*TPS1 and (*E,E*)-FDP, as well as between *Hh*CSP5 and (3*S*,6*S*,7*R*,10*S*)-10,11-epoxy-1-bisabolen-3-ol, (3*R*,6*S*,7*R*,10*S*)-10,11-epoxy-1-bisabolen-3-ol. All calculations were carried out in Discovery Studio (Accelrys, San Diego, CA, USA) Version 2.1.

## 3. Results

### 3.1. Identification of Key FDP and TPS Genes in the Aggregation Pheromone Biosynthesis Pathway

**Figure 2:**
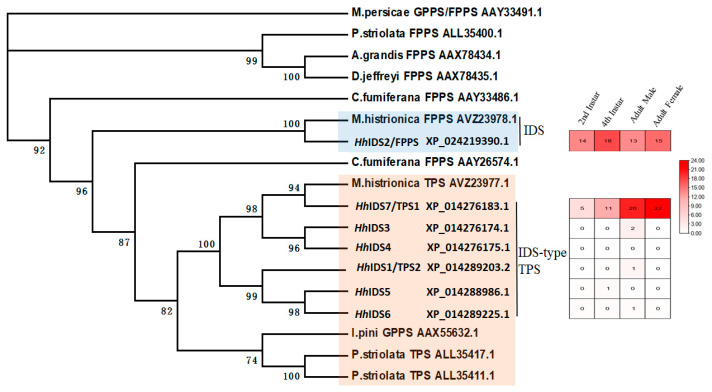
Phylogenetic tree of IDS genes from *H. halys* and other insects. Maximum likelihood phylogenetic tree was constructed by MEGA6.0 with the bootstrap value set to 1000 and the cut-off value set to 50%. TPM values of each IDS gene were calculated using transcriptomes of different developmental stages, sexes, and tissues of *H. halys*.

Through homology comparison, we identified seven IDS genes from the genome of *H. halys*, and a phylogenetic analysis showed that the *Hh*IDS2 and FDP genes of the harlequin bug (Genbank ID:AVZ23978.1) were in the same clan (Figure 2). The protein sequence homology of these two genes was 90%. The *Hh*IDS1 and *Hh*IDS3-7 genes were in the same clan as the TPS gene of the harlequin bug (Genbank ID:AVZ23977.1), and the *Hh*IDS7 gene was in the same subclan as the TPS gene of the harlequin bug (Figure 2). The protein sequence homology of these two genes was 80%. 

The OrthoVenn2 was used to identify orthologous genes among *H. halys* and *M. histrionica*, and a total of 19,589 orthologous clusters were identified (Table S2). Among these clusters, only two IDS genes were detected that were shared between *H. halys* and *M. histrionica.* One orthologous cluster was XP_014276183.1 (*Hh*IDS7) in *H. halys* and GECQ01420512.1 (TPS, Genbank ID:AVZ23977.1) in *M. histrionica*. Another orthologous cluster was XP_024219390.1(*Hh*IDS2) in *H. halys* and GECQ01414919.1 (FDP, Genbank ID:AVZ23978.1) *M. histrionica* (Table S2). According to the sequence homology (>99%) of these IDS genes with that of recently reported genes (*Hh*TPS1 Genbank ID: MG870387; *Hh*TPS2 Genbank ID: MG917093 and *Hh*FPPS Genbank ID: MG870389) [29], the *Hh*IDS7, *Hh*IDS1 and *Hh*IDS2 genes were named as *Hh*TPS1, *Hh*TPS2 and *Hh*FPPS, respectively. The results of expression profile analysis showed that *Hh*TPS2 and *Hh*IDS3-6 were almost not expressed in the second instar, fourth instar, adult male, and adult female of *H. halys*, while *Hh*FPPS was expressed in the second instar, fourth instar, adult male, and adult female with a TPS value between 13 and 18, with the highest expression level in the fourth instar. *Hh*TPS1 was also expressed in the second instar, fourth instar, adult male, and adult female with a TPS value between 6 and 22, and with the highest level of expression in the adult male and adult female (Figure 2).

Based on the above evolutionary relationship analysis and expression analysis, we concluded that *Hh*TPS1 is a key TPS gene for the synthesis of sesquipiperitol as a precursor for the aggregation pheromone in *H. halys*. *Hh*TPS1 may be responsible for catalyzing (*E,E*)-FDP generation (1*S*,6*S*,7*R*) sesquipiperitol. *Hh*FPPS is the FDP gene responsible for catalyzing IPP and DMAPP generation (*E,E*)-FDP. Both *Hh*FPPS and *Hh*TPS1 are key enzyme encoding genes in the aggregation pheromone biosynthesis pathway of *H. halys*.

### 3.2. Co-Expression Network Analysis to Identify the HhTPS1 Co-Expressed Genes

RNA sequencing was used to analyze the gene expression partners of all *H. halys* genes across the 15 samples types studied. Samples were clustered based on their gene expression level. As shown in Figure S1, the clustering results based on average distance and a hierarchical clustering algorithm showed that the materials used for sequencing had high repeatability in each process and the gene expression patterns in the same process were similar and clustered together. After screening the soft valve values (β), we selected the β value of nine. A weighted gene co-expression network was successfully constructed by the WGCNA method, and the network was divided into 19 modules. Among these modules, the TPS gene was detected in the red module, which suggests that the module may participate in the aggregation pheromone biosynthesis and action process of this pest. The number of genes in the red module was 1185 (Table S3). KEGG functional enrichment analysis of the genes of this module found that these genes were enriched in 25 pathways, including metabolic pathways, oxidative phosphorylation, carbon metabolism, peroxisome, and metabolism of xenobiotics by cytochrome P450 pathways, among others (Table S4). GO functional enrichment analysis showed that these genes were enriched in 76 GO terms, including the mitochondrion, mitochondrial respiratory chain complex I, mitochondrial electron transport, NADH to ubiquinone, plasma membrane, NADH dehydrogenase activity, and transferase activity, and that for transferring hexosyl groups, among others. (Table S5).

Further analysis of the red module (Table S3) identified candidate genes that may be involved in the regulation and recognition of aggregation pheromones in *H. halys* (Table 1). We identified seven P450 genes, including three CYP6a family members, three CYP6j family members, and one CYP4C family member. It is worth noting that these seven genes may be responsible for aggregation pheromone downstream biosynthesis. Seventeen transcription factors were also identified, including ten zinc finger proteins, two coiled-coil-helix-coiled-coil-helix domain-containing proteins, one putative homeodomain transcription factor, one RING finger protein, and three other types of transcription factors. These transcription factors may be responsible for the regulation of the expression of FDP, TPS, and P450 genes in the aggregation pheromone biosynthesis pathway. In addition, two olfactory-related genes were identified: one chemosensory protein 5 (*Hh*CSP5) and one odorant receptor 85b (*Hh*Or85b) (Table 1).

### 3.3. Molecular Docking of HhTPS1 and HhCSP5

From the above list of candidate genes, *Hh*TPS1 and *Hh*CSP5 were chosen for docking analysis. The template Human Fdps Synthase (PDB ID: 2rah) was selected to model the *Hh*TPS1 protein. Three models were constructed and obtained for *Hh*TPS1, of which the model with lowest DOPE was selected. After further analysis, the Ramachandran favored of the model was 94.66%, the ERRAT and Score was 96.2675 and the Verfy_3D (the ratio of residues in the model have an averaged 3D-1D) score was 80%. These results show that the results obtained by modeling are reasonable. Molecular docking analysis showed that (*E,E*)-FDP could be catalyzed by *Hh*TPS1, and the highest binding energy was −3.76468 kcal/mol, while the highest interaction energy was −56.368 kcal/mol. Molecular interaction analysis showed that there were Alkyl forces between three amino acids (LYS216, CYS275, and PRO460), while (*E,E*)-FDP, amino acids ARG132, ASP258 and LYS272 were linked by conventional hydrogen bonds to *Hh*TPS1 and (*E,E*)-FDP (Figure S2).

**Figure 3:**
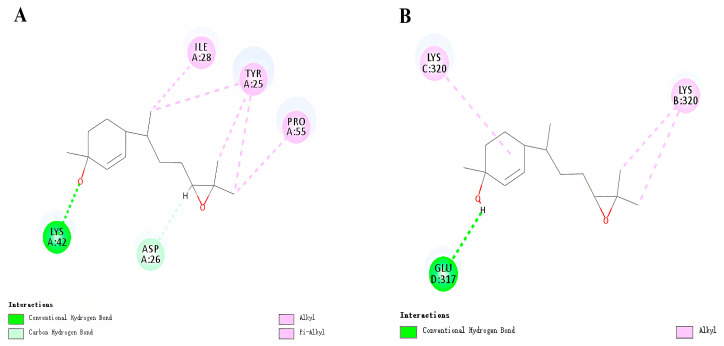
Molecular docking schematic of *Hh*CSP5 docking.Diagram of the interactions of (3*S*,6*S*,7*R*,10*S*)-10,11-epoxy-1-bisabolen-3-ol and (3*R*,6*S*,7*R*,10*S*)-10,11-epoxy-1-bisabolen-3-ol with key binding site residues of *Hh*CSP5 (**A**,**B**). Residues indicated in the figure have a distance to chemicals of <0.1 Å. Conventional hydrogen bonds and carbon hydrogen bonds are indicated in bright green and light green dashed lines, respectively. Alkyl and Pi-alkyls interactions are indicated in pink lines.

For *Hh*CSP5, the template CSP4 from *Schistocerca gregaria* (Forskal, 1775) (PDB ID: 2gvs) was selected to model the homology. The Ramachandran favored, ERRAT and Verfy_3D Score values for the model were 96.08%, 97.8495, and the 99.04%. Molecular docking analysis indicated that both (3*S*,6*S*,7*R*,10*S*)-10,11-epoxy-1-bisabolen-3-ol and (3*R*,6*S*,7*R*,10*S*)-10,11-epoxy-1-bisabolen-3-ol could be bound by *Hh*CSP5. The highest binding energy between *Hh*CSP5 and (3*S*,6*S*,7*R*,10*S*)-10,11-epoxy-1-bisabolen-3-ol was 6.62479 kcal/mol, and the highest interaction energy was −22.6357 kcal/mol. The amino acids LYS42 and ASP26 had conventional hydrogen bonds and carbon–hydrogen bonds to *Hh*CSP5 and (3*S*,6*S*,7*R*,10*S*)-10,11-epoxy-1-bisabolen-3-ol, respectively. Alkyl and Pi-Alkyl forces existed between three amino acids (TYR25, ILE28, and PRO55) and (3*S*,6*S*,7*R*,10*S*)-10,11-epoxy-1-bisabolen-3-ol (Figure 3A).

The highest binding energy and highest interaction energy between *Hh*CSP5 and (3*R*,6*S*,7*R*,10*S*)-10,11-epoxy-1-bisabolen-3-ol was 5.2411 and −22.0535 kcal/mol, respectively. There was Alkyl force between LYS320 of *Hh*CSP5 and (3*R*,6*S*,7*R*,10*S*)-10,11-epoxy-1-bisabolen-3-ol, and there was a conventional hydrogen bond between GLU317 and the (3*R*,6*S*,7*R*,10*S*)-10,11-epoxy-1-bisabolen-3-ol (Figure 3B). These amino acids were the key amino acid residues of the interaction between *Hh*CSP5 and aggregation pheromone.

## 4. Discussion

The aggregation pheromone of *H. halys* consists of sesquiterpenes, which play an important role in the ecology of *H. halys* [5,6]. However, these compounds have a complex structure and are an enantiomer mixture of two sesquiterpene alcohols, with a low chemical synthesis yield and potential for serious environmental pollution. Therefore, although pheromone-baited traps are effective in attracting *H. halys*, the cost of their production is too high for practical use. The average annual cost of using *H. halys* aggregation pheromone traps in the United States is about $900 US dollars per acre over the season [5]. The cost of chemical synthesis of aggregation pheromones in *H. halys* limits the large-scale application of this pheromone trapping technology. 

Compared with these costs of chemical synthesis, biosynthesis technology can potential to greatly reduce the cost of this aggregation pheromone, making the large-scale application of this pheromone feasible. Currently, the biosynthesis of a variety of sesquiterpenes through yeast cells, transgenic plants, or other technologies has been successfully implemented [46,47].

Biosynthesis technology is an effective tool to solve the problem of the high cost of chemical synthesis of the aggregation pheromone of *H. halys*. In this study, we identified the genes for the key enzymes (*Hh*FPPS and *Hh*TPS1) in the aggregation pheromone biosynthesis pathway, which have an orthologous relationship with the FDP and trans-IDS-type TPS genes of the aggregation pheromone biosynthesis pathway in the harlequin bug, with more than 80% protein sequence homology. Combined with expression profiling and molecular docking results, *Hh*TPS1 was further confirmed as the key enzyme gene for the biosynthesis of aggregation pheromones in *H. halys* (Figure 2 and Figure S2). The *in vitro* synthase activity assay of the *Hh*FPPS and *Hh*TPS1 genes has recently been confirmed [29]. The identification of these genes is useful for future potential large-scale biosynthesis of the aggregation pheromone of *H. halys* using metabolic engineering techniques, which would make widespread application of this pheromone inexpensive. In the future, the in vivo function of the *Hh*TPS1 gene should be further confirmed using RNAi, and CRISPR/Cas9. 

Through molecular docking methods, the key amino acid sites of *Hh*TPS1 that catalyze (*E,E*)-FDP were identified (Figure S2). The potentially important roles of ARG132, ASP258, and LYS272 in the interaction between *Hh*TPS1 and (*E,E*)-FDP have been supported by a recent study [29]. These amino acid sites are conducive to our understanding of the molecular interaction between *Hh*TPS1 and (*E,E*)-FDP. In the future, the directional evolution of *Hh*TPS1 enzymes could be realized through mutation of these amino acid sites to obtain the synthases with more catalytic activity and efficiency.

Through co-expression analysis, seven relevant P450 genes were identified (Table 1). These P450 genes are candidate genes responsible for the production of aggregation pheromones by P450 epoxidase catalyzation of sesquipiperitol or zingiberenol in the aggregation pheromone biosynthesis pathway [12,20]. Whether these reactions are catalyzed by a single or two separate P450 epoxidases remains to be determined [12]. So far, the P450 genes in the aggregation pheromone biosynthesis pathway of *H. halys* are still unknown and have not been reported in other stink bug species, such as harlequin bug *M. histrionica*, although some candidate P450 genes have been predicted in *M. histrionica* [21]. The P450 genes identified in this study are therefore candidate genes for understanding the downstream biosynthesis of the aggregation pheromone of *H. halys* and its relatives. These candidate genes can be validated by strategies such as RNAi, CRISPR/Cas9, or yeast or insect-cell expression. In the future, *Hh*TPS1 and P450 co-expression by *Saccharomyces cerevisiae* might allow useful strains to be engineered for large scale production. Such an engineered fungal strain could be used in the industrial production of the aggregation pheromone.

The transcription factors identified in this study may be involved in the regulation of the *Hh*FPPS, *Hh*TPS1 or *Hh*P450 genes in the aggregation pheromone biosynthesis pathway of *H. halys.* The function of these transcription factors needs to be verified by RNAi, gene editing, yeast-one-hybrid assays, or electrophoretic mobility shift assays. Based on the importance of aggregation pheromones in the behavior regulation of *H. halys*, these transcription factors are important candidate target genes for the future management and control of *H. halys* by gene editing or RNAi technology.

In the current study, we also identified a chemosensory protein and an olfactory receptor in *H. halys* that may recognize the aggregation pheromones. For these two candidate genes, in vivo functional studies can be carried out through RNAi or gene editing, followed by assays on the physiology or behavior responses of *H. halys* using the electroantennography technique and Y-tube olfactometers. For in vitro functional studies, the fluorescence-based binding assay could be conducted for *Hh*CSP5, and for *Hh*Or85b, we recommend use of the two-electrode voltage-clamp recording (in *Xenopus oocyte*) expression system.

In this study, we also detected the key amino acids of *Hh*CSP5 that bind to the two main components of the aggregation pheromones. The interaction between this chemosensory protein (*Hh*CSP5) and the aggregation pheromone is useful to design and develop precise environmentally friendly behavior regulation substances through a reverse chemical ecology approach to control *H. halys* populations. This method has been successfully applied in the screening of a variety of insect behavior-modifying substances [48,49,50]. Using this method, we anticipate being able to obtain *H. halys* behavior-regulating substances that might have similar functions to the natural aggregation pheromone while being cheaper to synthesize and more stable during use. The docking analysis of the interaction between *Hh*Or85b and the aggregation pheromone was not carried out, because we could not detect an appropriate template with reasonable homology. In insects, the recognition of pheromones often involves multiple odorant-binding proteins and chemoreceptors [49,51,52,53]. Therefore, in addition to the *Hh*CSP5 identified in this study, there may be other OBPs or CSPs responsible for the recognition and transport of aggregation pheromones in *H. halys*.

In conclusion, we identified *Hh*TPS1, a candidate enzyme for aggregation pheromone biosynthesis in *H. haly*s, and we also found some P450s, transcriptional factors, and olfactory-related genes that are co-expressed with *Hh*TPS1. Altogether, these genes provide a solid foundation for understanding the biosynthesis pathways and recognition mechanisms of aggregation pheromones in *H. haly*s. These genes also provide key candidate targets for the management and control of *H. halys* through CRISPR/Cas9 or RNAi technology.

## Figures and Tables

**Table 1:** Candidate genes co-expressed with HhTPS1 identified by WGCNA analysis.

Potential Function	Coexpressed Genes	Weight Values	Annotation
Synthase genes in aggregation pheromone biosynthesis pathway	XM_024359849.1	0.030	cytochrome P450 6a14 (LOC106686595)
	XM_014425704.2	0.080	cytochrome P450 6j1 (LOC106683935)
	XM_014435358.2	0.068	cytochrome P450 6a2-like (LOC106690077)
	XM_024361356.1	0.128	cytochrome P450 6j1-like (LOC106678797)
	XM_024361357.1	0.184	cytochrome P450 6j1-like (LOC106678797)
	XM_024362540.1	0.176	cytochrome P450 6a2-like (LOC106678035)
	XM_014428854.1	0.092	cytochrome P450 4C1 (LOC106685872)
Transcriptional factors in aggregation pheromone biosynthesis pathway	XM_024361675.1	0.040	zinc finger protein 711-like (LOC106678825)
	XM_024362041.1	0.211	zinc finger MIZ domain-containing protein 1-like (LOC106691938)
	XM_024359956.1	0.177	zinc finger FYVE domain-containing protein 1-like (LOC106682986)
	XM_024360254.1	0.175	putative homeodomain transcription factor (LOC106692479)
	XM_014438343.2	0.11	zinc finger BED domain-containing protein 4-like (LOC106692406)
	XM_014438440.2	0.206	RING finger protein 37 (LOC106692477)
	XM_014438969.1	0.039	transcriptional regulatory protein AlgP (LOC106692793)
	XM_014433039.2	0.083	transcription factor kayak (LOC106688534)
	XM_014433901.1	0.055	zinc finger autosomal protein-like (LOC106689113)
	XM_014435086.2	0.108	zinc finger autosomal protein-like (LOC106689884)
	XM_014435137.2	0.215	zinc finger protein 711-like (LOC106689915)
	XM_014430570.2	0.054	zinc finger protein 420 (LOC106686950)
	XM_014431700.2	0.137	coiled-coil-helix-coiled-coil-helix domain-containing protein 1 (LOC106687684)
	XM_014425878.2	0.058	coiled-coil-helix-coiled-coil-helix domain-containing protein 2-like (LOC106684044)
	XM_014427582.2	0.053	zinc finger MYM-type protein 1 (LOC106685089)
	XM_014428786.2	0.077	zinc finger protein 629-like (LOC106685840)
	XM_014432021.2	0.222	transcription factor A, mitochondrial (LOC106687869)
Recognition and transport aggregation pheromones	XM_024358492.1	0.178	odorant receptor 85b-like (LOC106692425)
	XM_014421632.2	0.124	chemosensory protein 5 [*Nezara viridula*] (LOC106681357)

## Data Availability

The data presented in this study are available in Supplementary Materials.

## Supplementary Materials

The following supporting information can be downloaded at: https://www.mdpi.com/article/10.3390/insects14020163/s1, Table S1: SRA numbers and description used in this study; Table S2: Orthologous gene clusters between *Halyomorpha halys* and *Murgantia histrionica*; Table S3: Detail information in red module; Table S4: KEGG function enrichment result of red module; Table S5: GO function enrichment result of red module. Figure S1. Sample clustering by WGCNA. Figure S2. Molecular docking schematic of *Hh*TPS1 docking.
