# Supplementary figures and images for: Exploration of Candidate Genes Involved in the Biosynthesis, Regulation and Recognition of the Male-Produced Aggregation Pheromone of Halyomorpha halys
Source: Insects. 2023 Feb 8;14(2):163. doi: 10.3390/insects14020163 (PMC9960045; doi:10.3390/insects14020163)

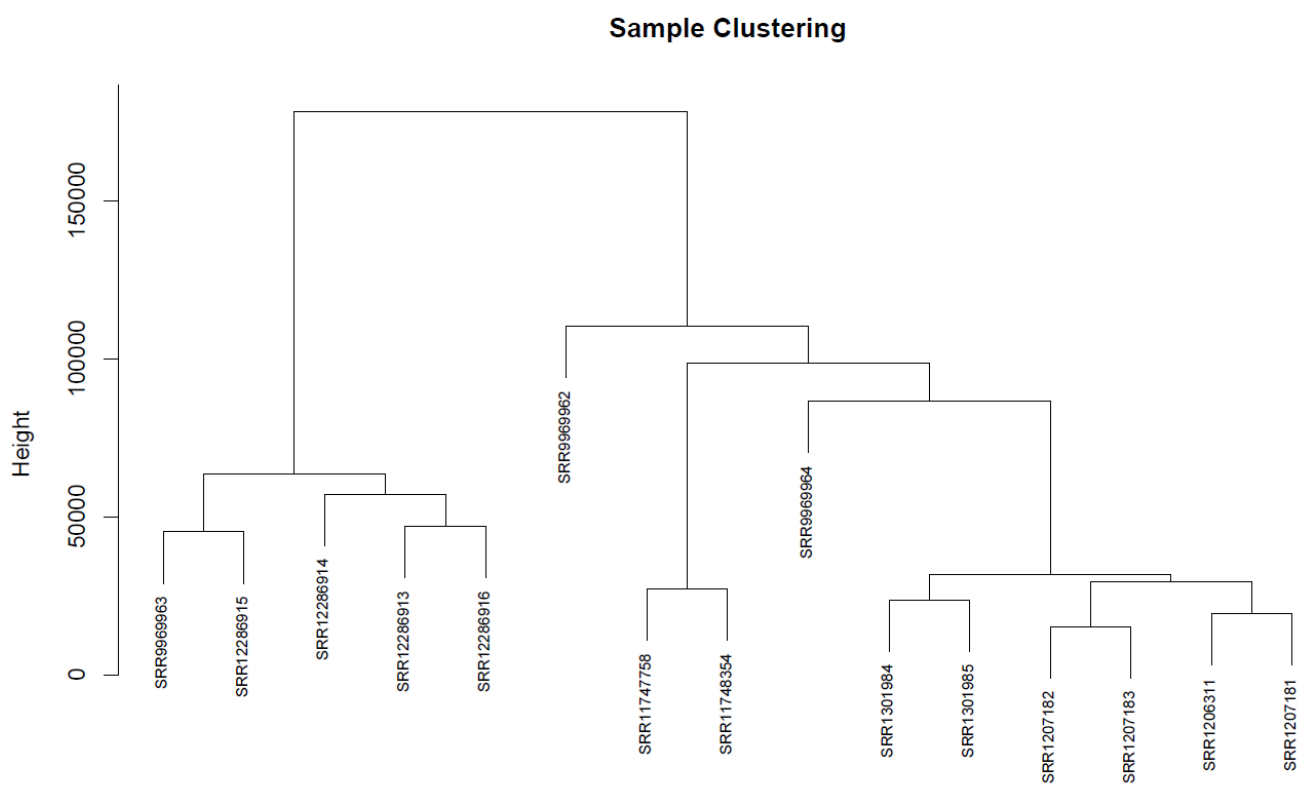

**Figure S1.** Sample clustering by WGCNA.

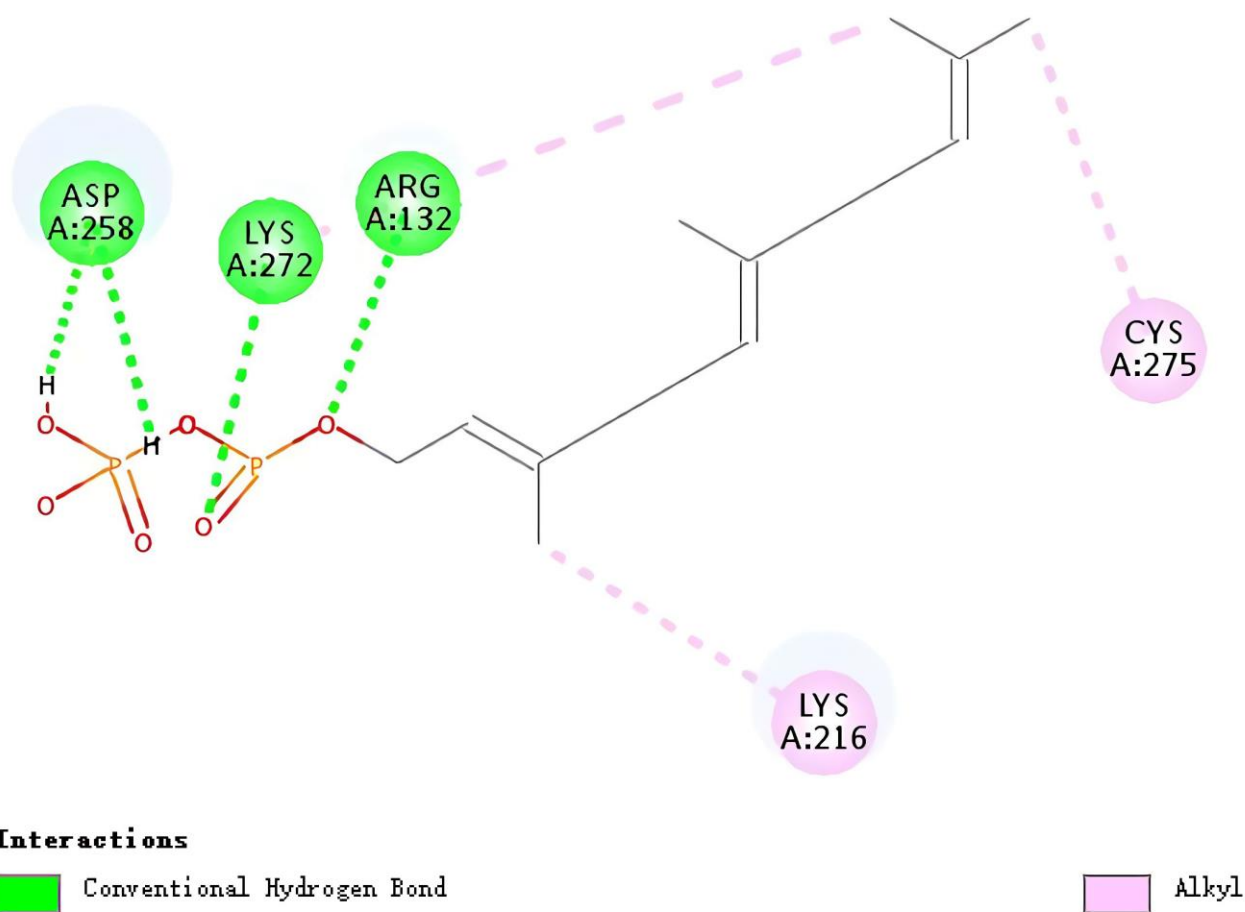

Figure S2. Molecular docking schematic of *HhTPS1* docking.

Supplement: Supplementary file 1 [file insects-14-00163-s001.zip › supplementary figures.pdf]
